# CD4+ T cell expression of the IL-10 receptor is necessary for facial motoneuron survival after axotomy

**DOI:** 10.1186/s12974-020-01772-x

**Published:** 2020-04-17

**Authors:** Elizabeth M. Runge, Abhirami K. Iyer, Deborah O. Setter, Felicia M. Kennedy, Virginia M. Sanders, Kathryn J. Jones

**Affiliations:** 1 Department of Anatomy, Cell Biology, and Physiology, Indiana University School of Medicine, 635 Barnhill Drive, Medical Science Building 5035, Indianapolis, IN 46202 USA; 2 Research and Development, Richard L. Roudebush VA Medical Center, Indianapolis, IN USA; 3 Department of Neuroscience, Washington University School of Medicine, St. Louis, MO USA; 4 Department of Cancer Biology and Genetics, The Ohio State University, Columbus, OH USA

**Keywords:** Autoimmune, Axotomy, IL-10, Motoneuron, Nerve injury, Neuroprotection, T cell

## Abstract

**Background:**

After peripheral nerve transection, facial motoneuron (FMN) survival depends on an intact CD4+ T cell population and a central source of interleukin-10 (IL-10). However, it has not been determined previously whether CD4+ T cells participate in the central neuroprotective IL-10 cascade after facial nerve axotomy (FNA).

**Methods:**

Immunohistochemical labeling of CD4+ T cells, pontine vasculature, and central microglia was used to determine whether CD4+ T cells cross the blood-brain barrier and enter the facial motor nucleus (FMNuc) after FNA. The importance of IL-10 signaling in CD4+ T cells was assessed by performing adoptive transfer of IL-10 receptor beta (IL-10RB)-deficient CD4+ T cells into immunodeficient mice prior to injury. Histology and qPCR were utilized to determine the impact of IL-10RB-deficient T cells on FMN survival and central gene expression after FNA. Flow cytometry was used to determine whether IL-10 signaling in T cells was necessary for their differentiation into neuroprotective subsets.

**Results:**

CD4+ T cells were capable of crossing the blood-brain barrier and associating with reactive microglial nodules in the axotomized FMNuc. Full induction of central IL-10R gene expression after FNA was dependent on CD4+ T cells, regardless of their own IL-10R signaling capability. Surprisingly, CD4+ T cells lacking IL-10RB were incapable of mediating neuroprotection after axotomy and promoted increased central expression of genes associated with microglial activation, antigen presentation, T cell co-stimulation, and complement deposition. There was reduced differentiation of IL-10RB-deficient CD4+ T cells into regulatory CD4+ T cells in vitro.

**Conclusions:**

These findings support the interdependence of IL-10- and CD4+ T cell-mediated mechanisms of neuroprotection after axotomy. CD4+ T cells may potentiate central responsiveness to IL-10, while IL-10 signaling within CD4+ T cells is necessary for their ability to rescue axotomized motoneuron survival. We propose that loss of IL-10 signaling in CD4+ T cells promotes non-neuroprotective autoimmunity after FNA.

## Background

Maintenance of central motoneuron survival is paramount in order for functional recovery to be achieved following peripheral nerve transection and disconnection from target musculature. After facial nerve axotomy (FNA), immunodeficient mice lacking B and T cells exhibit more facial motoneuron (FMN) death in the facial motor nucleus (FMNuc) compared to wild-type (WT) mice with intact immune systems [1, 2]. In the FNA model of peripheral nerve injury, the neuroprotective capacity of the adaptive immune system is specific to Th2 CD4+ T cells, which are sufficient for rescuing FMN survival when adoptively transferred into immunodeficient RAG-2^-/-^ mice [3, 4]. The precise mechanism of CD4+ T cell-mediated neuroprotection is unknown, but recent research shows that the peripheral immune system plays a major role in modulating the central glial response to facial nerve injury [5].

Naïve CD4+ T cells must encounter major histocompatibility complex (MHC) class II on antigen presenting cells (APCs) in the periphery and become activated in an antigen-specific fashion in order to become neuroprotective [6]. After peripheral activation and clonal expansion, T cells are next attracted to the FMNuc by centrally derived chemokines [7–9]. It has been inferred from immunohistochemical (IHC) studies demonstrating the presence of CD3+ T lymphocytes in axotomized FMNuc that these cells cross the blood-brain barrier (BBB) [10–12]. However, further evidence utilizing histological labeling of the FMNuc vasculature is needed to strengthen the hypothesis that T cells (and the CD4+ T subset in particular) exist in CNS parenchyma after axotomy and are not confined to penetrating blood vessels, which are difficult to visualize without appropriate labeling techniques. After homing to the injured FMNuc, T cells must again encounter MHC class II on microglia in order to confer neuroprotection [6]. Microglia proliferate in the FMNuc after axotomy [13], phagocytize dead neurons [14], and express MHC class I and II molecules, on which they may present neuronal antigen to infiltrating T cells [15, 16].

The anti-inflammatory cytokine interleukin-10 (IL-10) has received considerable attention in neuroinflammation research due to its modulatory effects on glial reactivity and pro-survival influences on neurons [17, 18]. IL-10 is an important mediator of the neuroprotective capability of CD4+ T cells after FNA; in the absence of IL-10, axotomy induces greater FMN death that is resistant to rescue by CD4+ T cells. Yet, despite the necessity of both IL-10 and CD4+ T cells for neuroprotection after FNA, CD4+ T cells are not a requisite source of IL-10. IL-10 likely derives from CNS resident cells in the FMNuc, rather than CD4+ T cells. Although absence of CD4+ T cells results in slightly decreased IL-10 levels in the FMNuc at an early time point after axotomy, this modest difference is unlikely to wholly explain the survival deficit observed in RAG-2^-/-^ mice at 1 month post-operation [19]. It has not been elucidated previously whether the dual actions of IL-10- and CD4+ T cell-mediated neuroprotection after FNA are interrelated or independent of one another. This information is necessary to understand the process of endogenous immune-mediated neuroprotection and thereby identify potential mechanisms that can be exploited for the treatment of nerve injury and motoneuron disease.

For the first part of this study, it was hypothesized that CD4+ T cells play an integral role in the central IL-10 cascade after FNA by regulating IL-10 receptor (IL-10R) expression in the FMNuc. The ability of peripheral CD4+ T cells to regulate central gene expression in the axotomized FMNuc has been previously established [5]. Prior to testing this hypothesis, it was necessary to determine whether T cells must communicate with cells in the FMNuc remotely (i.e., across the BBB) or are capable of traveling into the FMNuc parenchyma and interacting directly with resident cells there. This information would indicate whether central expression of IL-10R and other markers are potentially effected by direct T cell contact.

The second part of this study was performed to investigate IL-10R expression by infiltrating T cells themselves. IL-10 directly induces T cell tolerance to specific antigen [20]. Furthermore, it has been shown that tolerized T cells can inhibit further antigen presentation events and suppress the proliferation of other T cells in an antigen-specific fashion [21, 22]. Conversely, effector T cells (particularly Th1 and Th17 cells) can activate APCs (including microglia) to a pro-inflammatory state [23–26]. Therefore, an IL-10 cascade in the axotomized FMNuc could reflect a mechanism regulating autoimmune responses to neuronal self-antigen released after FNA. We propose that IL-10R expression on CD4+ T cells is necessary for their ability to confer neuroprotection, potentially by influencing T cell activation against self-antigen and subsequent modulation of the glial microenvironment by T cells after axotomy.

## Methods

Animals: all mice were obtained from The Jackson Laboratory. Strains utilized include C57BL/6 J (WT), B6(Cg)-*Rag2*^*tm1.1Cgn*^/J (RAG-2^-/-^), and B6.129S2-*Il10rb*^*tm1Agt*^/J (*Il10rb*^-/-^). Only female mice were used due to the tendency of males to engage in fighting behavior after axotomy, leading to infections at the surgery site that complicate immune profiles. Animals were obtained at 7 weeks of age and allowed to acclimate for at least 1 week prior to use. Mice were housed in sterilized microisolator cages equipped with a laminar flow system to maintain a pathogen-free environment and allowed access to autoclaved food pellets and water ad libitum.

Adoptive transfer: donor animals were utilized at a 1:1 ratio to recipients. Following CO_2_ euthanasia and cervical dislocation, spleens were removed and mechanically dissociated using the plunger of a sterile syringe. Dissociated splenocytes were incubated with CD4 magnetic beads (Miltenyi Biotec, 130-117-043) and manually sorted using LS columns (Miltenyi Biotec, 130-042-401) following manufacturer instructions. The eluent was then passed through a new column a second time to enhance cell purity. Flow cytometry revealed sorted CD4+ T cell purity to be 96% (data available upon request). Host mice received 5 million sorted CD4+ T cells per 100 μl PBS injection via tail vein 1 week prior to FNA to allow for engraftment of transferred cells.

Surgical procedure: FNA was performed when mice were 8 weeks old. The detailed surgical procedure has been published previously [27]. Briefly, animals were anesthetized with 2.5% isoflurane, and a small (~ 4 mm) incision was made posterior to the right ear protuberance using aseptic techniques. The underlying fascia was dissected bluntly to expose the facial nerve, which was severed just distal to its emergence from the stylomastoid foramen. The nerve stumps were either pushed away, or a small segment of the distal nerve stump was resected to prevent reconnection. The left facial nerve was left intact as an internal control. Following surgery, successful transection of the facial nerve was confirmed by behavioral assessment of eye blink and whisking reflexes.

Immunohistochemistry: The 14 days post-operation (dpo) time point was selected for analysis as this represents the peak of T cell infiltration in the literature [11, 16]. Animals (*n* = 3) were euthanized via ketamine-xylazine overdose and exsanguination followed by perfusion with 2% buffered paraformaldehyde (PFA). Brains were removed, post-fixed in 2% PFA for 1–3 h, and equilibrated in 30% sucrose prior to embedding in OCT medium. Eight micrometer brainstem sections containing the FMNuc were collected and blocked for 1 h at room temperature in 10% donkey serum, 1% bovine serum albumin, and 0.01% Triton X-100 in PBS, followed by incubation in primary antibody (Table 1) for 4 h at room temperature or 16 h at 4 °C. Sections were washed 3 × 5 min in PBS prior to incubation with secondary antibody for 1 h at room temperature. When fluorescent Nissl staining was desired, NeuroTrace™ 435/455 Blue Fluorescent Nissl Stain (Thermo Fisher, N21479) was diluted 1:100 in PBS, applied to sections for 20 min after removing secondary antibody, and washed 3 × 5 min in PBS prior to mounting in ProLong™ Gold Antifade medium (Invitrogen, P36930). Images were captured with an Olympus BX50 inverted fluorescent microscope using the Olympus cellSens Entry 1.9 software, and level adjustments to reduce background were performed uniformly across control and axotomized FMNuc in Adobe Photoshop.

**Table 1:** Antibodies utilized for IHC

	Antibody	Manufacturer and Cat. no.	Dilution
**Primary**	Rabbit anti-IBA1	Thermo Fisher 019-19741	1:500
	Rat anti-CD4 488	BioLegend 100423	1:100
	Rabbit anti-CD3	Abcam ab16669	1:200
	Rabbit anti-CD31	Abcam ab124432	1:500
**Secondary**	Donkey anti-rabbit 568 (used for IBA1, CD3, CD31)	Abcam ab175470	1:1000
	Donkey anti-rat 488 (used for CD4)	Thermo Fisher A21208	1:1000

Motoneuron counting: animals (*n* = 4–5/group) were euthanized at 28 dpo using CO_2_ inhalation and cervical dislocation. Brains were removed and flash-frozen at the interface of a pre-chilled 37.5% 2-methylbutane/62.5% 1-bromobutane biphasic solution prior to cryosectioning. Brainstem sections spanning the caudal-rostral extent of the FMNuc were collected at 25 μm, fixed for 15 min in 4% PFA, and stained with 0.04% thionin acetate solution followed by ethanol dehydration series. Sections were cleared in CitriSolv overnight or up to 3 days and subsequently coverslipped using Permount toluene-based mounting medium. For counting, an impartial investigator coded all slides. A separate blinded investigator used a Leica DMRB light microscope and Neurolucida software (version 10.31) to manually count motoneurons in the FMNuc. To avoid double counting, only FMN profiles with a nucleus and nucleolus were counted. Mean percentage FMN survival was quantified by dividing the total number of FMN on the axotomized side by the total number on the control side and multiplying by 100%. One-way analysis of variance (ANOVA) followed by a Student-Newman-Keuls post-hoc test was performed with an alpha of 0.05.

Laser capture microdissection, RNA extraction, reverse transcription (RT), and qPCR: *N* = 4-11 mice/group were analyzed at the 14 dpo time point according to the rationale described in previous experiments; additionally, this time point represents the peak of microglial gene expression in the FMNuc after axotomy [5]. All procedures were performed as described in Setter et al. [5]. Briefly, brains were removed, flash-frozen, and sectioned onto PEN membrane slides (Leica Microsystems, 11505158). Slides were stained with thionin, and a Leica ASLMD microscope was used to collect the right and left FMNuc. RNA extraction and RT were performed using an Arcturus PicoPure® RNA Isolation Kit (Applied Biosystems, KIT0204) and SuperScript® VILO Master Mix (Invitrogen, 11755050), respectively. TaqMan® FAM gene expression assays were used to perform qPCR (Table 2). CT values were normalized to the reference gene *Gapdh* and fold changes in mRNA expression between axotomized and control FMNuc were calculated. Because *Tnf* is only expressed on the axotomized FMNuc, the ΔΔCT value was calculated using a spleen cDNA standard, and its levels in axotomized FMNuc are expressed relative to WT spleen. The same spleen standard was used for comparison across all samples. Outliers were detected using the Grubbs’ test (GraphPad QuickCalcs). One-way ANOVA followed by Student-Newman-Keuls multiple comparisons was performed with an alpha of 0.05 to determine significance.

**Table 2:** TaqMan® assays utilized in this study

Gene	TaqMan ID	RefSeq accession number
*B2m*	Mm00437762_m1	NM_009735.3
*C3*	Mm01232779_m1	(GenBank–no RefSeq numbers available) BC029976.1, BC043338.1, DQ408205.1, EU868829.1, HM856604.1, K02782.1
*Cd40*	Mm00441891_m1	NM_011611.2, NM_170702.2, NM_170703.2, NM_170704.2, NR_027852.1
*Cd86*	Mm00444543_m1	NM_019388.3
*Gapdh*	Mm99999915_g1	NM_001289726.1, NM_008084.3
*H2ab1*	Mm00439216_m1	NM_207105.3
*Nos2*	Mm00440502_m1	NM_010927.3
*Tnf*	Mm00443260_g1	NM_013693.3

Cell culture: anti-CD3e (BD Biosciences, 553057) at 10 μg/mL concentration in sterile PBS was incubated on 6-well flat-bottom tissue culture plates overnight at 4 °C. Spleens (*n* = 3/group) were dissociated in sterile FACS buffer, subjected to RBC lysis, and resuspended in RPMI 1640 supplemented with 10% heat inactivated FBS, 2 mM l-glutamine, 50 μM β-mercaptoethanol, and 1% penicillin/streptomycin. 4–6 × 10^6^ cells were deposited per well on pre-incubated and washed plates (stimulated cells) or PBS-only plates (unstimulated cells). Two micrograms per milliliter of anti-CD28 (BD Biosciences, 553294) was added to the stimulated cells, and plates were incubated overnight at 37 °C. The next day, cells were stimulated further with 10 ng/ml PMA and 1000 ng/ml ionomycin for 4–6 h. Brefeldin A was added at 10 μg/ml for the last 3 h to all wells (both stimulated and unstimulated).

**Fig. 1:**
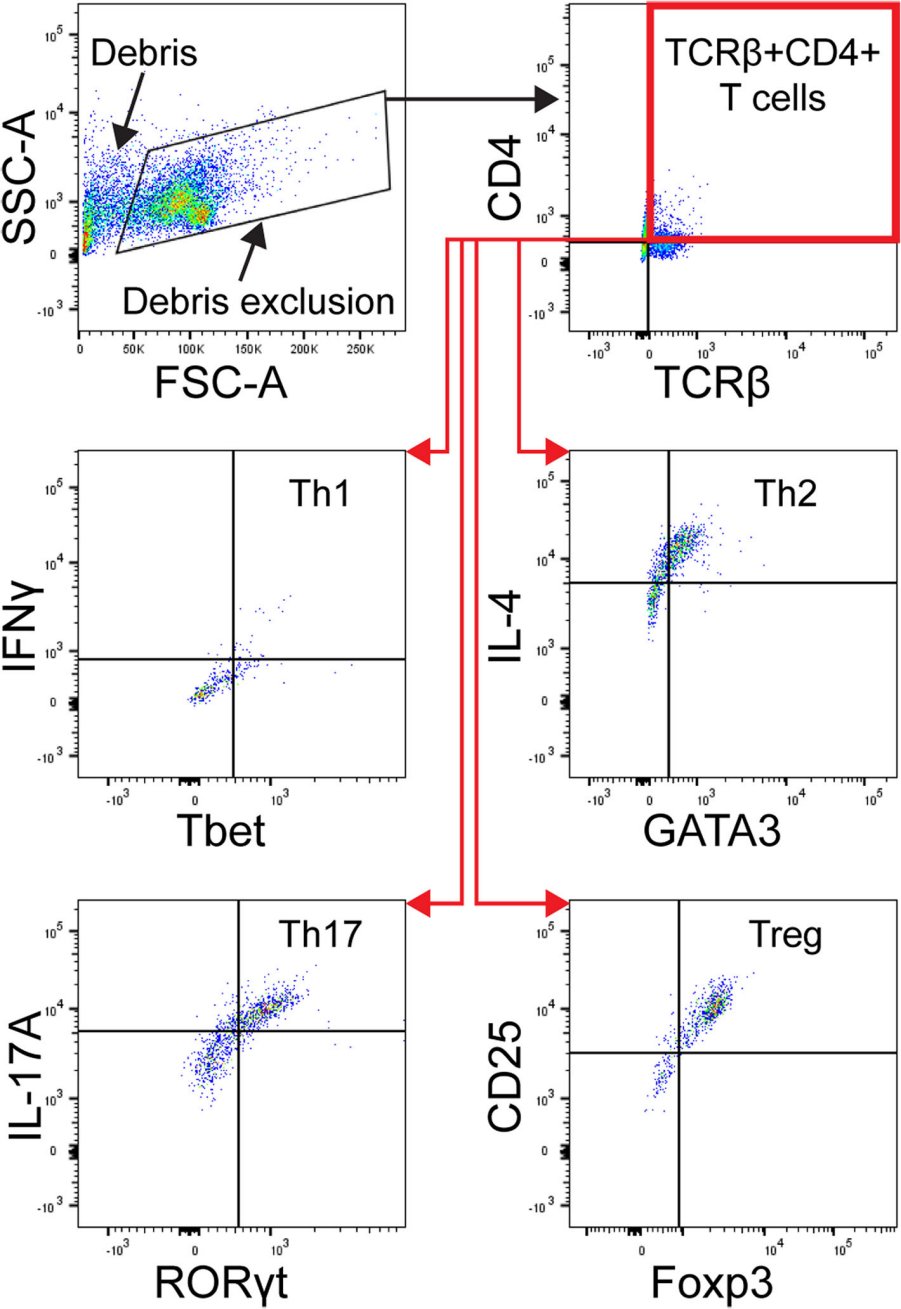
Diagram depicting representative gating strategies for delineating CD4+ T cell subset populations. Following gating for singlets and debris exclusion, the CD4+ T cell population was selected based on CD4 and TCRβ expression. Within this population, the Th1 subset was defined as TBET^+^IFNγ^+^, Th2 as GATA3^+^IL-4^+^, Th17 as RORγt^+^IL-17A^+^, and regulatory T (Treg) helper subset as CD25^high^FOXP3^+^

Flow cytometry: cells were harvested and blocked with anti-CD16/CD32 at 1:1000 in FACS buffer for 15 min before washing and staining for surface markers TCRβ and CD4 for 30 min on ice. Cells were washed, fixed in 1% PFA for 15 min, and washed again prior to permeabilization in FACS buffer + 0.1% saponin for 15 min at room temperature. Antibody cocktails for intracellular staining were mixed in FACS buffer +0.1% saponin and applied to cells for 30 min at room temperature. Antibody information can be found in Table 3. Cells were washed, resuspended in FACS buffer, and stored at 4 °C until flow cytometry was performed (within 1 day). Data was acquired using a Fortessa X20 flow cytometer. A minimum number of 10,000 events were collected using the BD FACSDiva software. FCS files were analyzed using FlowJo 10. Cells were first gated for singlets, and CD4+ T cells and downstream T cell helper subset populations were defined following the representative gating strategies depicted in Fig. 1. Comparison of cell populations was performed in GraphPad Prism using one-way ANOVA followed by Student-Newman-Keuls multiple comparisons with an alpha of 0.05 to determine significance.

**Table 3:** Antibodies utilized for flow cytometry

Antibody	Manufacturer and cat. no.	Dilution
APC anti-mouse TCR β chain	BioLegend 109212	1:50
Alexa Fluor 488 anti-mouse CD4	BioLegend 100423	1:800
BV 421 anti-mouse CD4	BioLegend 100437	1:50
BV 711 anti-mouse IL-4	BioLegend 504133	1:50
BV 421 anti-mouse IFN-γ	BioLegend 505829	1:50
BV 711 anti-mouse IL-17A	BioLegend 506941	1:50
BV 711 anti-mouse CD25	BioLegend 102049	1:50
PE anti-GATA3	BioLegend 653803	1:50
PE/Dazzle™ 594 anti-T-bet	BioLegend 644827	1:50
PE anti-RORγt	Invitrogen AFKJS-9	1:50
Alexa Fluor 488 anti-mouse/rat/human FOXP3	BioLegend 320011	1:100
Purified anti-mouse CD16/32	BioLegend 101310	1:1000

## Results

It is unknown whether the CD4+ T cells responsible for mediating neuroprotection after axotomy are capable of infiltrating into the CNS parenchyma, or alternatively whether they must communicate with CNS resident cells across the BBB. Previous work using single-label IHC has reported the presence of CD3+, CD4+, and CD8+ cells in the FMNuc after axotomy [11, 16, 28–30]. However, given the limitations of single-marker IHC as well as the potential expression of CD4 on non-T cells, including microglia and macrophages [31], it was necessary to perform co-IHC of CD3 with CD4 to demonstrate that true CD4+ T cells infiltrate the FMNuc after FNA where they may potentially interact with centrally located cells.

**Fig. 2:**
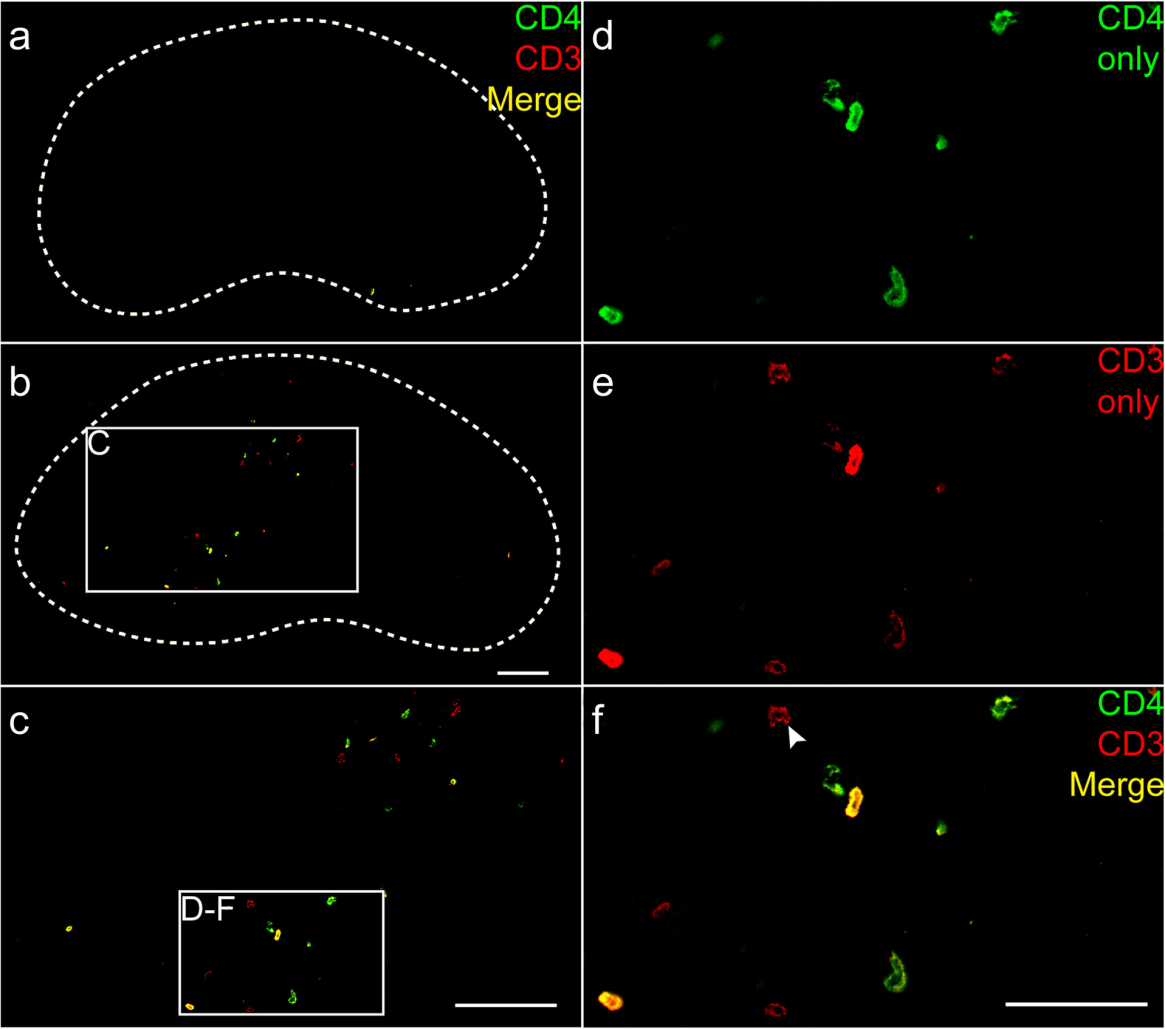
IHC identification of CD3 + CD4+ T cells in the FMNuc. **a** Control FMNuc at 14 dpo. White dashed line indicates boundary of FMNuc. **b** Low power image of axotomized FMNuc at 14 dpo with T cell infiltration. **c** Higher power image of boxed area in **b**. **d** High power image of boxed area in **c**, CD4 channel only (green). **e** Same field as **d** showing CD3 channel only (red). **f** Overlaid channels in **d** and **e** showing CD3+/CD4− (white arrowhead) and CD3+/CD4+ T cells. Scale bar in **b** = 100 μm and applies to **a** and **b**. Scale bar in **c** = 100 μm. Scale bar in **d** = 50 μm and applies to **d**–**f**

**Fig. 3:**
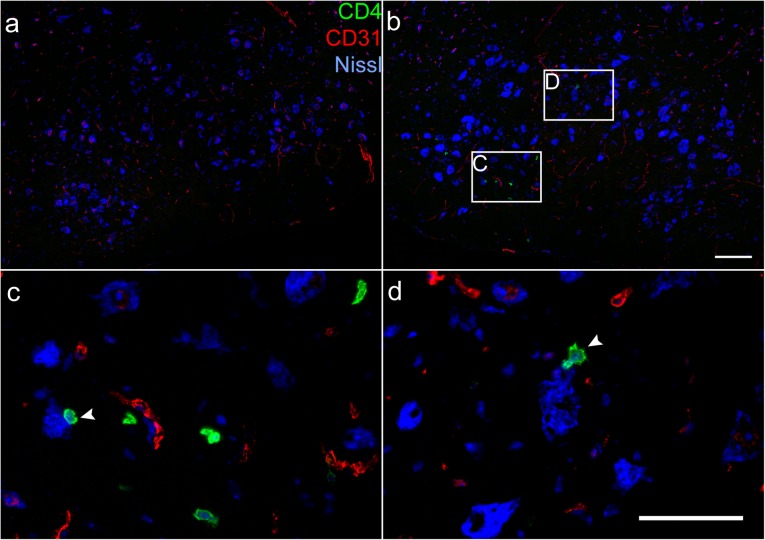
IHC of CD4+ T cells (green) and CD31-labeled endothelial layer of blood vessels (red) penetrating the FMNuc. **a** Composite image of control FMNuc at 14 dpo showing no infiltrating T cells. **b** Composite image of axotomized FMNuc at 14 dpo. **c**, **d** Magnified fields from boxes in **b** showing CD4+ T cells are not contained within CD31-labeled blood vessels. Note, CD4+ T cells marked with arrowheads in close proximity to FMN labeled with fluorescent Nissl stain (blue). Scale bar in **b** = 100 μm and applies to **a** and **b**. Scale bar in **d** = 50 μm and applies to **c** and **d**

Antibodies specific for CD3 and CD4 antigen revealed no infiltrate in the unoperated FMNuc (Fig. 2a). In the contralateral axotomized FMNuc, infiltrates of CD3+ and CD4+ cells were detected (Fig. 2b–f). In every tissue section analyzed, all CD4+ cells observed infiltrating the FMNuc were CD3+, but not every CD3+ T cell was CD4+, indicating that CD8+ T cells may also infiltrate the FMNuc after axotomy (Fig. 2d–f). Although CD4+ T cells were detected in the FMNuc after axotomy, this observation alone was not sufficient to determine whether they are capable of interacting directly with cells in CNS parenchyma, or whether they are restricted to the vascular compartment. In combination with anti-CD4 to label T cells and a fluorescent Nissl stain to label neurons, an antibody against CD31 was used to visualize vasculature in the FMNuc. This triple stain revealed the presence of small penetrating blood vessels in the control FMNuc without evidence of T cell infiltration (Fig. 3a). CD4+ T cells were detectable in the axotomized FMNuc at 14 dpo. These T cells appeared not to be contained within CD31-labeled blood vessels, indicating that they are capable of extravasating into the CNS parenchyma (Fig. 3b–d). Furthermore, some T cells appeared to be in close proximity to injured motoneurons (Fig. 3c, d, arrowheads).

**Fig. 4:**
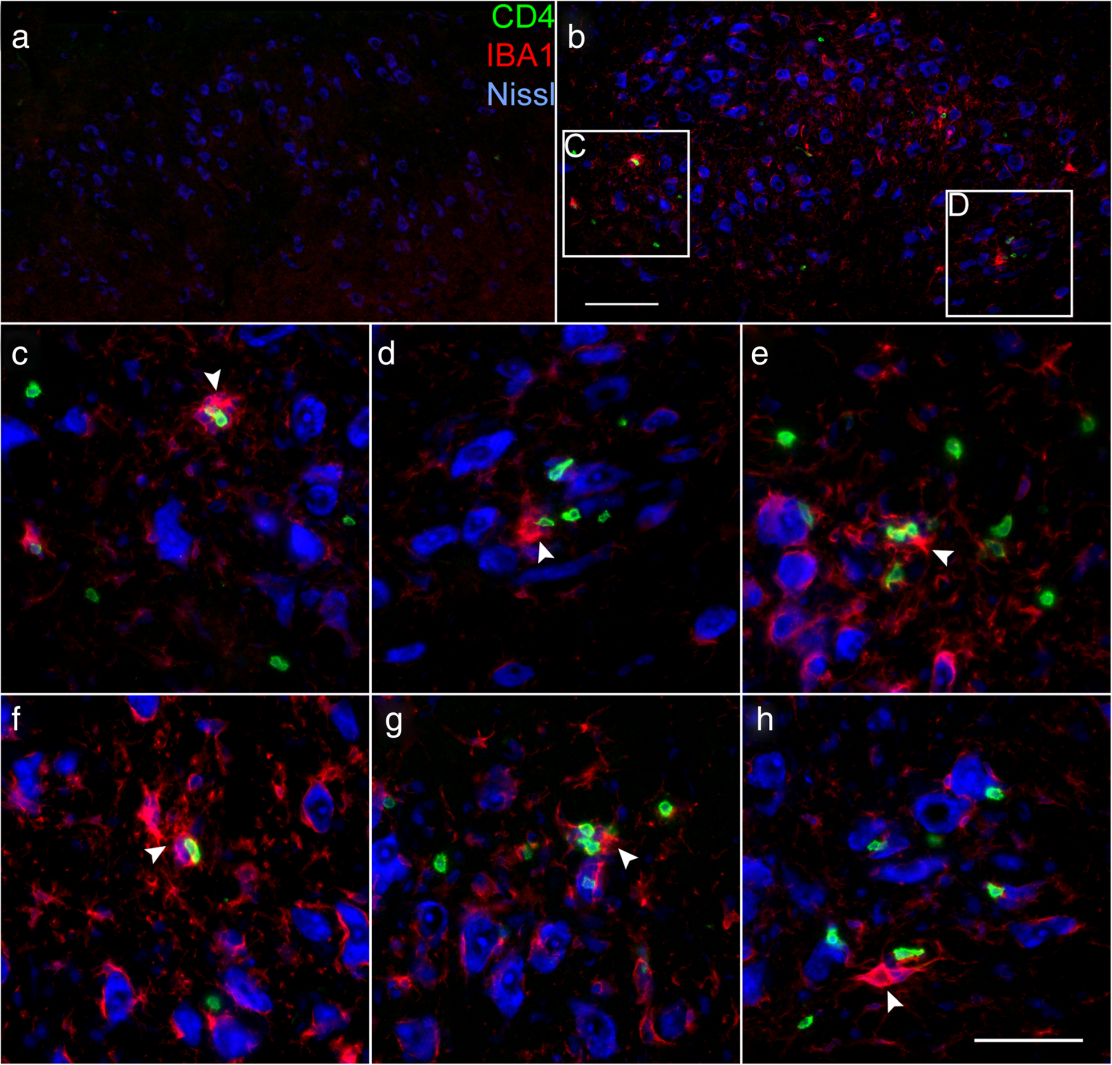
IHC of CD4+ T cells (green) and IBA+ microglia (red) in the FMNuc. Nissl-labeled neurons are shown in blue. **a** Composite image of control FMNuc at 14 dpo showing no infiltrating T cells or reactive IBA+ microglia. **b** Composite image of axotomized FMNuc at 14 dpo. **c**, **d** Magnified fields from boxes in **b** showing IBA1+ microglial nodules (arrowheads) with associated CD4+ T cells. **e**–**h** Representative fields from axotomized FMNuc in other experimental animals showing IBA1+ microglial nodules (arrowheads) with associated CD4+ T cells. Scale bar in **b** = 100 μm and applies to **a** and **b**. Scale bar in **h** = 50 μm and applies to **c**–**h**

CD4+ T cells must interact with MHC class II on a central APC to mediate neuroprotection after axotomy [6]. With the ability of CD4+ T cells to extravasate into the FMNuc parenchyma established, potential associations of CD4+ T cells with microglia were next evaluated. Double labeling for CD4 and IBA1 in the unaxotomized FMNuc was negative for reactive microglia or infiltrating CD4+ cells (Fig. 4a). At 14 dpo, microglia in the axotomized FMNuc became reactive as evidenced by increased expression of IBA1, and a CD4+ T cell infiltrate was observed (Fig. 4b). These CD4+ T cells often appeared to be clustered around reactive microglial nodules (Fig. 4c–h, arrowheads).

**Fig. 5:**
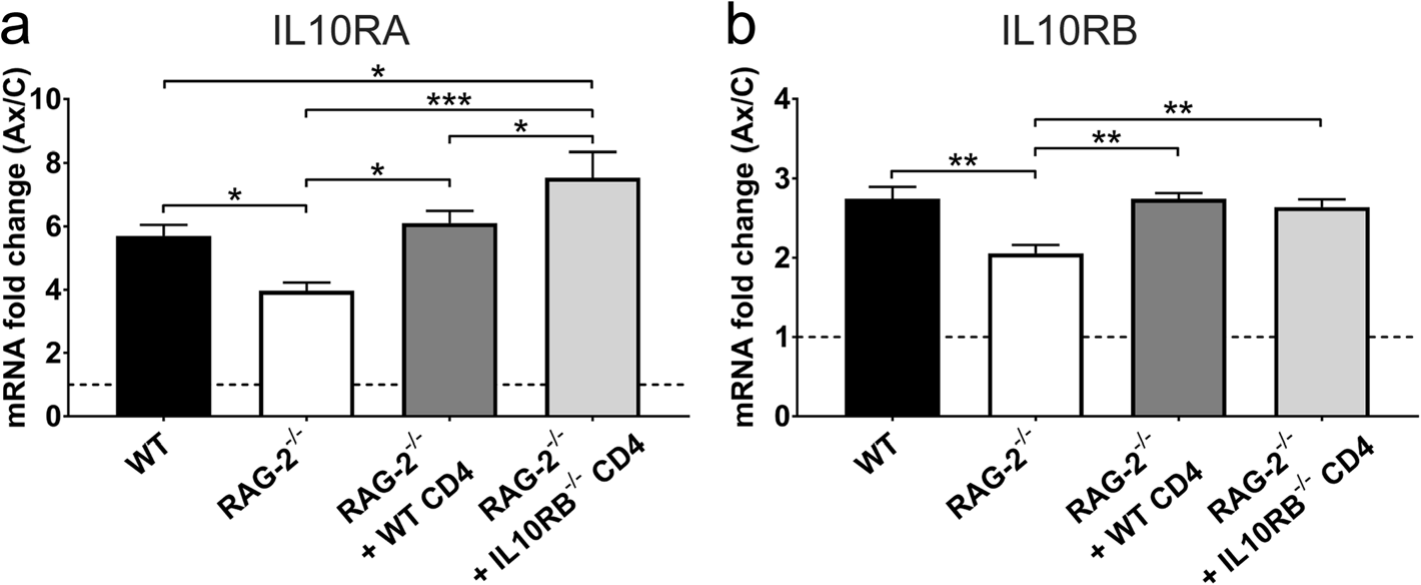
mRNA fold change of **a***Il10ra* and **b***Il10rb* in the axotomized FMNuc relative to control FMNuc at 14 days post-FNA in WT, RAG-2^-/-^, RAG-2^-/-^ + WT CD4+ T cells, and RAG-2^-/-^ + *Il10rb*^-/-^ CD4+ T cells. Bars represent mean fold change ± SEM. Dotted line represents baseline level of gene expression in control FMNuc. *p < 0.05, **p < 0.01, ***p < 0.001

To determine whether the adaptive immune system has an effect on central upregulation of the IL-10R after axotomy, qPCR was performed for IL-10R subunit gene expression in the FMNuc of WT, immunodeficient, and immune-cell reconstituted mice. Gene expression was calculated as fold change of mRNA expression in axotomized FMNuc relative to control FMNuc. Fold change in *Il10ra* expression (Fig. 5a) was significantly reduced in RAG-2^-/-^ (3.97 ± 0.26) compared to WT (5.70 ± 0.35, *p* < 0.05) and was restored to WT levels after adoptive transfer of WT CD4+ T cells (6.12 ± 0.38). Fold change in *Il10rb* expression (Fig. 5b) was likewise significantly reduced in RAG-2^-/-^ (2.06 ± 0.10) compared to WT (2.74 ± 0.15, *p* < 0.01), and adoptive transfer of WT CD4+ T cells restored normal *Il10rb* expression (2.74 ± 0.07). These data indicate that CD4+ T cells are necessary for full upregulation of IL-10R subunits in the FMNuc after axotomy.

This regulation of IL-10R expression by CD4+ T cells could be due to T cell-triggered induction of IL-10R expression on CNS resident cells and/or infiltration of IL-10R-expressing T cells into the FMNuc after axotomy. To elucidate which scenario occurs after FNA, RAG-2^-/-^ mice were reconstituted with CD4+ T cells lacking *Il10rb* expression, which is necessary for IL-10 signal transduction [32, 33]. *Il10ra* fold change in RAG-2^-/-^ mice reconstituted with *Il10rb*^-/-^ CD4+ T cells (Fig. 5a, 7.54 ± 0.65) was significantly increased relative to RAG-2^-/-^ (*p* < 0.001), indicating that IL-10 signaling within T cells was not necessary for T cells to regulate *Il10ra* gene expression in the FMNuc. In fact, upregulation of *Il10ra* in RAG-2^-/-^ mice given *Il10rb*^-/-^ CD4+ T cells was significantly greater than in WT or RAG-2^-/-^ given WT CD4+ T cells (*p* < 0.05), perhaps due to a compensatory reaction in the FMNuc. *Il10rb* fold change in RAG-2^-/-^ reconstituted with *Il10rb*^-/-^ CD4+ T cells (Fig. 5b, 2.64 ± 0.09) was significantly increased relative to RAG-2^-/-^ (*p* < 0.001) and was not significantly different from WT or RAG-2^-/-^ + WT CD4+ T cells. Due to absence of *Il10rb* expression by the transgenic T cells, this restoration of *Il10rb* gene expression could only be due to a T cell-mediated induction of *Il10rb* in CNS resident cells in the FMNuc.

**Fig. 6:**
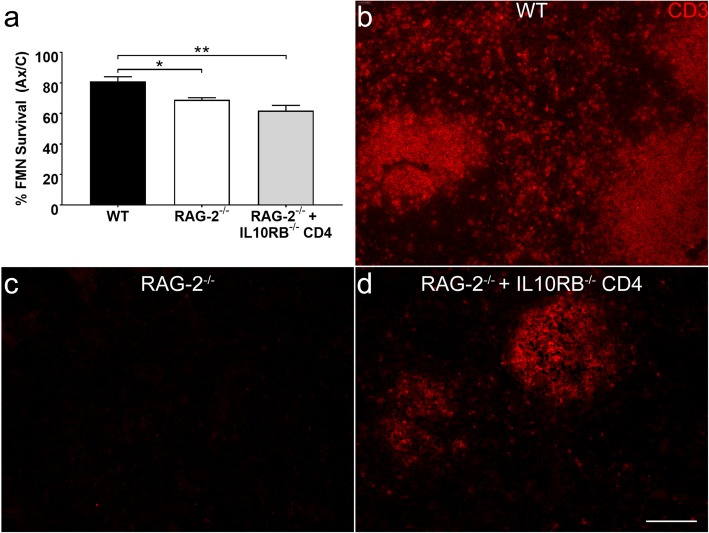
**a** Average percent survival of axotomized FMN relative to control ± SEM at 28 dpo in WT, RAG-2^-/-^, and RAG-2^-/-^ reconstituted with *Il10rb*^-/-^ CD4+ T cells. Survival in both RAG-2^-/-^ and RAG^-/-^ reconstituted with *Il10rb*^-/-^ CD4+ T cells was decreased relative to WT, indicating that *Il10rb*^-/-^ T cells fail to confer neuroprotection. *p < 0.05, **p < 0.01. **b** IHC showing CD3+ T cells in WT splenic follicles. **c** CD3+ cells are absent in RAG-2^-/-^ spleens. **d** Adoptive transfer of *Il10rb*^-/-^ CD4+ T cells restores CD3+ expression in splenic follicles, indicating that *Il10rb*^-/-^ CD4+ T cells were successfully transplanted and remain viable in vivo until 28 dpo. Scale bar = 100 μm and applies to **b**–**d**

Although it was found that *Il10rb*^-/-^ CD4+ T cells were capable of triggering an increase in IL-10R expression by CNS resident cells in the FMNuc, the neuroprotective capacity of these T cells remained unknown. It is well established in the literature that RAG-2^-/-^ mice exhibit decreased FMN survival after axotomy relative to WT, and that WT CD4+ T cells restore FMN survival in RAG-2^-/-^ mice to WT levels [2, 3]. The ability of *Il10rb*^-/-^ CD4+ T cells to mediate neuroprotection was assayed by quantifying FMN survival in WT, RAG-2^-/-^, and RAG-2^-/-^ reconstituted with *Il10rb*^-/-^ CD4+ T cells at 28 dpo. In accordance with the literature, survival in RAG-2^-/-^ was significantly decreased compared to WT (Fig. 6a, 69 ± 1.3% compared to 81 ± 3%, *p* < 0.05). However, *Il10rb*^-/-^ CD4+ T cells failed to rescue FMN survival; survival in reconstituted animals remained low compared to WT (61.8 ± 3.5%, *p* < 0.01). To confirm that adoptively transferred *Il10rb*^-/-^ T cells were still viable in vivo at 28 dpo, IHC was performed using spleens from WT, RAG-2^-/-^, and RAG-2^-/-^ mice reconstituted with *Il10rb*^-/-^ CD4+ T cells. WT spleens contained CD3+ T cell follicles that were absent in RAG-2^-/-^ spleen and restored in RAG-2^-/-^ given *Il10rb*^-/-^ CD4+ T cells (Fig. 6b, c), indicating that transferred cells successfully engrafted into host lymphoid tissue. Overall, these data suggest that the increase in IL-10R expression by CNS resident cells, although mediated by T cells, is insufficient for FMN survival after FNA; rather, IL-10R signaling by the T cell itself may be necessary for neuroprotection.

To investigate the role of T cell IL-10R signaling in modulating central APC activation after FNA, qPCR for genes associated with microglial activation, antigen presentation, T cell co-stimulation, and complement deposition/synaptic pruning was performed in the FMNuc of WT, RAG-2^-/-^, and RAG^-/-^ mice reconstituted with either WT or *Il10rb*^-/-^ CD4+ T cells. The hypothesis was that loss of IL-10 receptor signaling on T cells would promote a pro-inflammatory, autoimmune-reactive microglial/macrophage gene signature in the FMNuc.

**Fig. 7:**
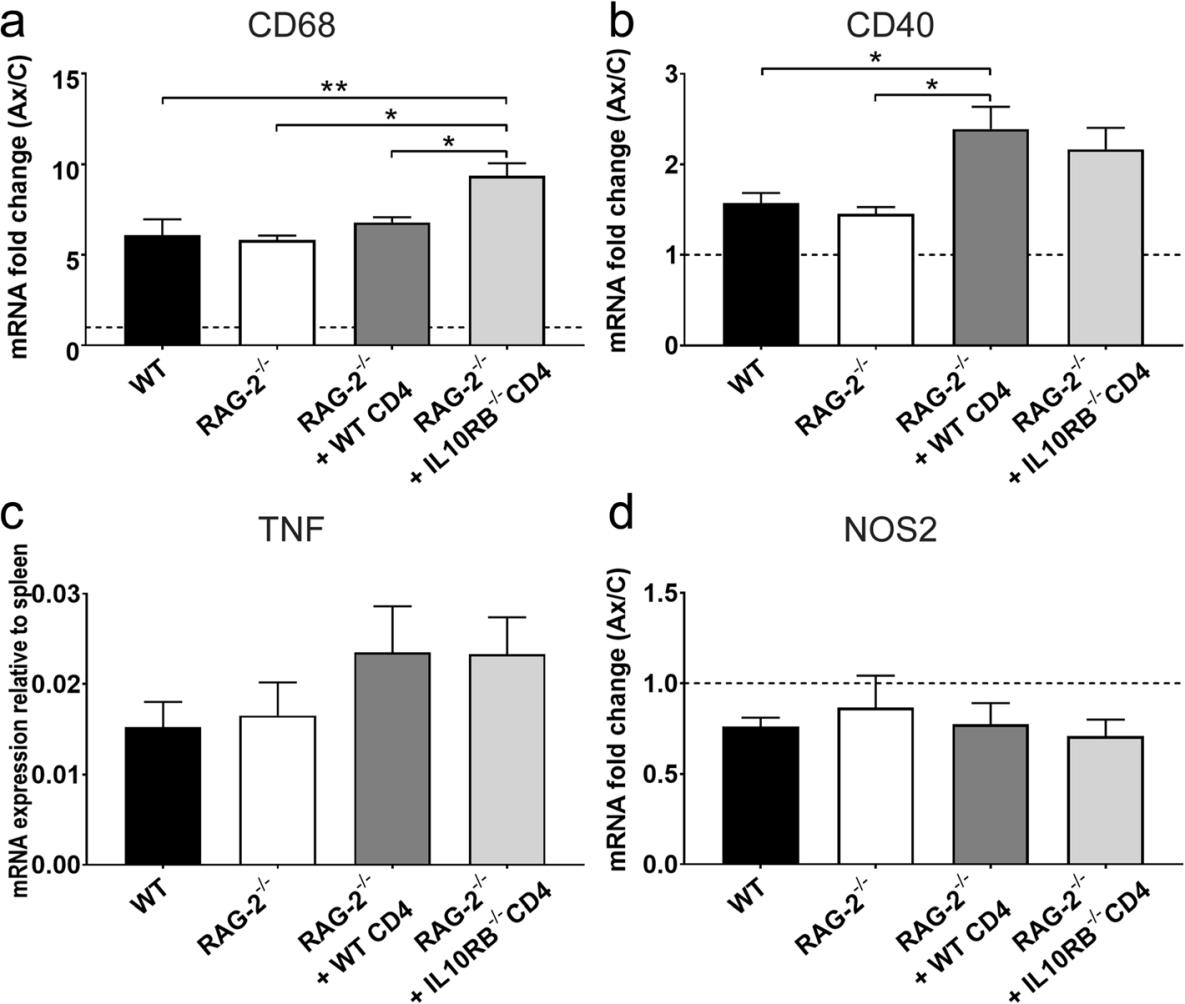
mRNA fold change of **a***CD68*, **b***Cd40*, **c***Tnf*, and **d***Nos2* in the axotomized FMNuc relative to control FMNuc (except for *Tnf*, which is relative to spleen control) at 14 days post-FNA in WT, RAG-2^-/-^, RAG-2^-/-^ + WT CD4+ T cells, and RAG-2^-/-^ + *Il10rb*^-/-^ CD4+ T cells. Bars represent mean fold change ± SEM. *p < 0.05, **p < 0.01

*Cd68*, *Cd40*, *Tnf*, and *Nos2* were genes selected for evaluation of general microglial pro-inflammatory activation. At 14 dpo, *Cd68* mRNA increased approximately sixfold in the WT axotomized FMNuc; there was no difference in *Cd68* expression between WT, RAG-2^-/-^, and RAG-2^-/-^ mice reconstituted with WT CD4+ T cells (Fig. 7a, 6.078 ± 0.88, 5.809 ± 0.25, 6.785 ± 0.29, respectively). RAG-2^-/-^ mice reconstituted with *Il10rb*^-/-^ CD4+ T cells had significantly increased *Cd68* expression in the axotomized FMNuc (9.37 ± 0.69) relative to WT (*p* < 0.01), RAG-2^-/-^, and RAG-2^-/-^ + WT CD4+ T cells (*p* < 0.05), indicating that *Il10rb*^-/-^ T cells promote an increase in general microglial reactivity after axotomy. *Cd40* mRNA was modestly increased in the axotomized FMNuc relative to control across all conditions, and there were no significant differences between WT, RAG-2^-/-^, and RAG-2^-/-^ + *Il10rb*^-/-^ CD4+ T cells (Fig. 7b, 1.57 ± 0.11, 1.46 ± 0.08, 2.17 ± 0.24, respectively). There was also no significant difference between RAG-2^-/-^ mice reconstituted with WT CD4+ T cells (2.40 ± 0.25) and those reconstituted with *Il10rb*^-/-^ CD4+ T cells, although the former showed significantly increased *Cd40* expression relative to WT and RAG-2^-/-^ (*p* < 0.05).

No *Tnf* transcript was detected in the control FMNuc, in agreement with previous studies [5, 34]. Therefore, to compare changes in *Tnf* mRNA expression, the differences between *Tnf* and *Gapdh* cycle threshold values were normalized to the same standardized spleen control for each axotomized FMNuc sample. As shown in Fig. 7c, *Tnf* mRNA in axotomized FMNuc at 14 dpo ranged between 1–3% of spleen standard, and no differences were observed between WT, RAG-2^-/-^, RAG-2^-/-^ + WT CD4+ T cell, and RAG-2^-/-^ + *Il10rb*^-/-^ CD4+ T cell groups (0.015 ± 0.003, 0.017 ± 0.004, 0.024 ± 0.005, 0.023 ± 0.004, respectively). *Nos2* mRNA did not appear to be upregulated in the axotomized FMNuc and in fact may have slightly decreased relative to control in WT and RAG-2^-/-^ mice receiving *Il10rb*^-/-^ CD4+ T cells (Fig. 7d, results of one-sample *t* test against fold change of 1: WT, 0.76 ± 0.05, *p* = 0.008; RAG-2^-/-^ + *Il10rb*^-/-^ CD4, 0.71 ± 0.09, *p* = 0.033). There were no significant differences detected between these groups and RAG-2^-/-^ mice or RAG-2^-/-^ + WT CD4+ T cells (0.87 ± 0.17 and 0.78 ± 0.11, respectively).

**Fig. 8:**
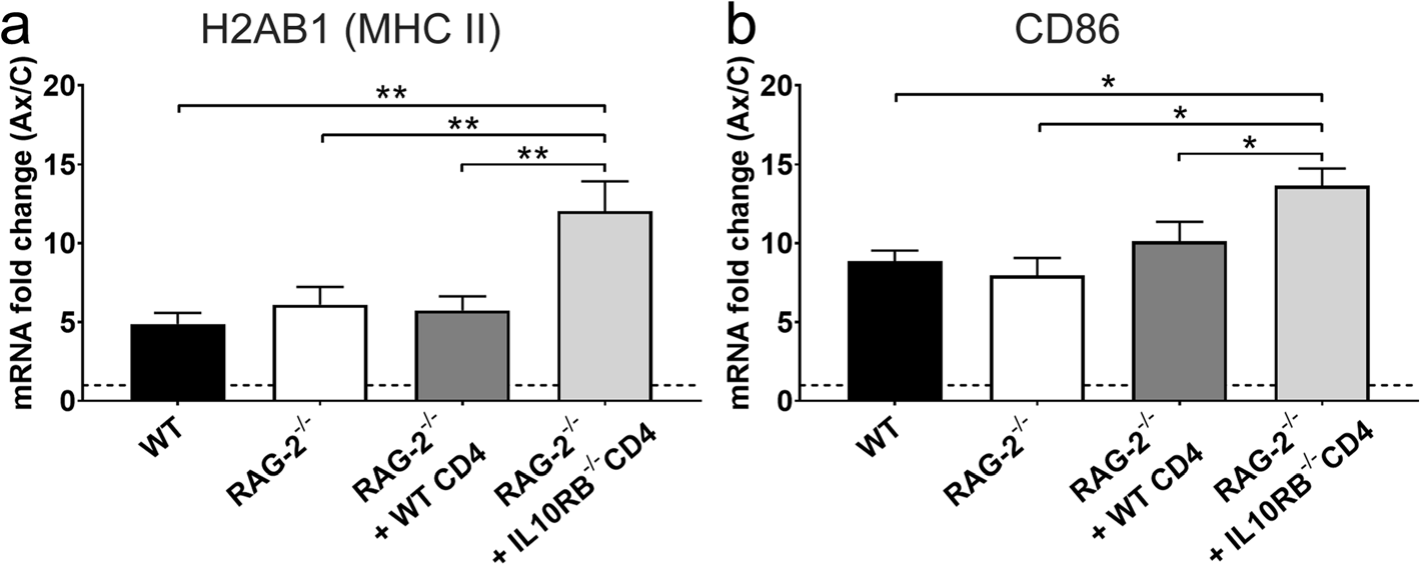
mRNA fold change of **a***H2ab1* and **b***Cd86* in the axotomized FMNuc relative to control FMNuc at 14 days post-FNA in WT, RAG-2^-/-^, RAG-2^-/-^ + WT CD4+ T cells, and RAG-2^-/-^ + *Il10rb*^-/-^ CD4+ T cells. Bars represent mean fold change ± SEM. *p < 0.05, **p < 0.01

*H2ab1* and *Cd86* were selected for analysis of microglial antigen presentation and T cell co-stimulation-associated gene expression in the axotomized FMNuc. *H2ab1* encodes the predominant MHC class II haplotype expressed in C57BL/6 J mice, and CD86 is a co-stimulatory molecule expressed by microglia that interacts with CD28 on T cells. Axotomy robustly induced *H2ab1* in WT animals; expression increased approximately fivefold relative to control FMNuc (Fig. 8a). RAG-2^-/-^ mice that received adoptive transfer of *Il10rb*^-/-^ CD4+ T cells exhibited dramatically higher induction of *H2ab1* expression after axotomy compared to WT, RAG-2^-/-^, and RAG-2^-/-^ + WT CD4+ T cells (12.5 ± 1.88 vs. 4.9 ± 0.71, 6.1 ± 1.12, 5.8 ± 0.89, respectively; *p* < 0.01). Expression of *Cd86* also increased robustly in response to axotomy with approximate 9-fold induction in WT (Fig. 8b). RAG-2^-/-^ mice given *Il10rb*^-/-^ CD4+ T cells exhibited significantly increased expression of *Cd86* after axotomy compared to WT, RAG-2^-/-^, and RAG-2^-/-^ + WT CD4+ T cells (13.7 ± 1.10 vs. 8.9 ± 0.67, 7.9 ± 1.13, 10.1 ± 1.24, respectively, *p* < 0.05).

**Fig. 9:**
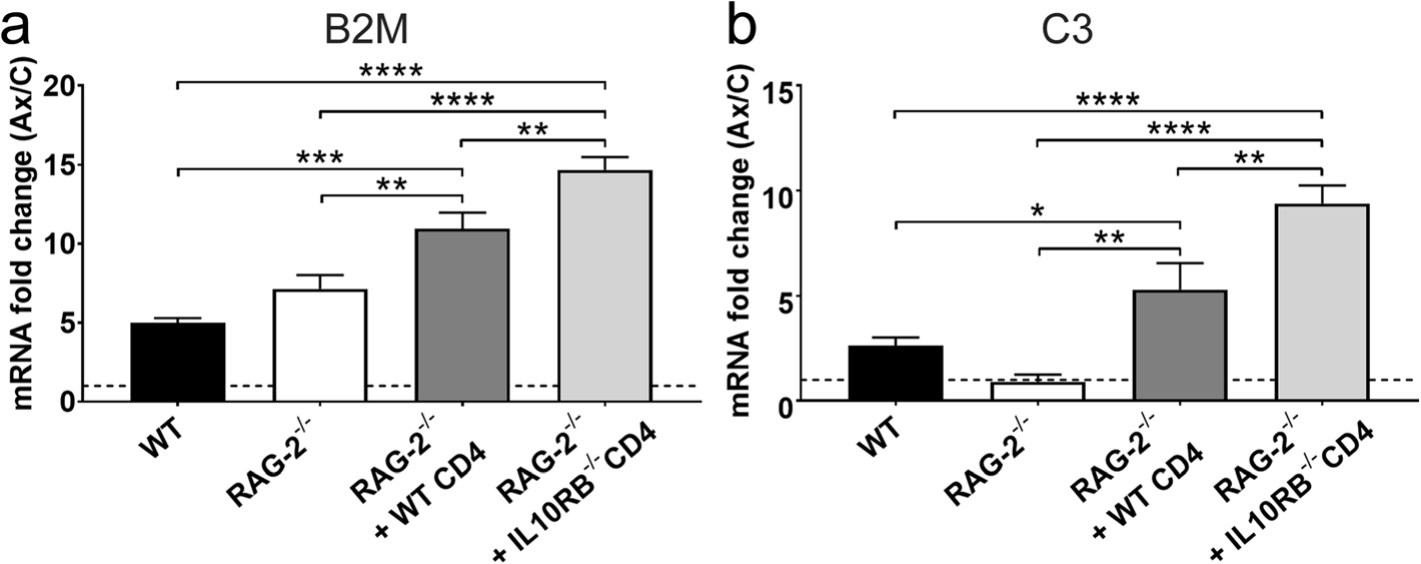
mRNA fold change of **a***B2m* and **b***C3* in the axotomized FMNuc relative to control FMNuc at 14 days post-FNA in WT, RAG-2^-/-^, RAG-2^-/-^ + WT CD4+ T cells, and RAG-2^-/-^ + *Il10rb*^-/-^ CD4+ T cells. Bars represent mean fold change ± SEM. *p < 0.05, **p < 0.01, ***p < 0.001, ****p < 0.0001

Both MHC class I and C3 are associated with microglial phagocytic nodule formation and synaptic pruning [16, 35–37]. *B2m* encodes the MHC class I subunit β2-microglobulin. *B2m* mRNA expression increased fivefold in the WT axotomized FMNuc and was not significantly different in RAG-2^-/-^ mice (Fig. 9a, 5.0 ± 0.28, 7.2 ± 0.85, respectively). Adoptive transfer of WT CD4+ T cells into RAG-2^-/-^ mice resulted in significantly increased *B2m* expression relative to WT and RAG-2^-/-^ (RAG-2^-/-^ + WT CD4 11.0 ± 1.02 vs. WT, *p* < 0.001; vs. RAG-2^-/-^, *p* < 0.01). RAG-2^-/-^ mice given *Il10rb*^-/-^ CD4+ T cells had significantly increased expression of *B2m* after axotomy compared to WT, RAG-2^-/-^, and RAG-2^-/-^ + WT CD4+ T cells (RAG-2^-/-^ + *Il10rb*^-/-^ CD4 14.9 ± 0.81 vs. WT and RAG-2^-/-^, *p* < 0.0001; vs. RAG-2^-/-^ + WT CD4, *p* < 0.01). *C3* mRNA increased modestly in the FMNuc of WT mice after axotomy and was not significantly different in RAG-2^-/-^ mice (Fig. 9b, 2.6 ± 0.39, 0.9 ± 0.35, respectively). Adoptive transfer of WT CD4+ T cells into RAG-2^-/-^ mice resulted in increased *C3* expression (5.3 ± 1.24 vs. WT, *p* < 0.05; vs. RAG-2^-/-^, *p* < 0.01). Adoptive transfer of *Il10rb*^-/-^ CD4+ T cells resulted in even greater *C3* expression than in WT, RAG-2^-/-^, or RAG-2^-/-^ + WT CD4+ T cells (9.4 ± 0.86 vs. WT and RAG-2^-/-^, *p* < 0.0001; vs. RAG-2^-/-^ + WT CD4, *p* < 0.01).

**Fig. 10:**
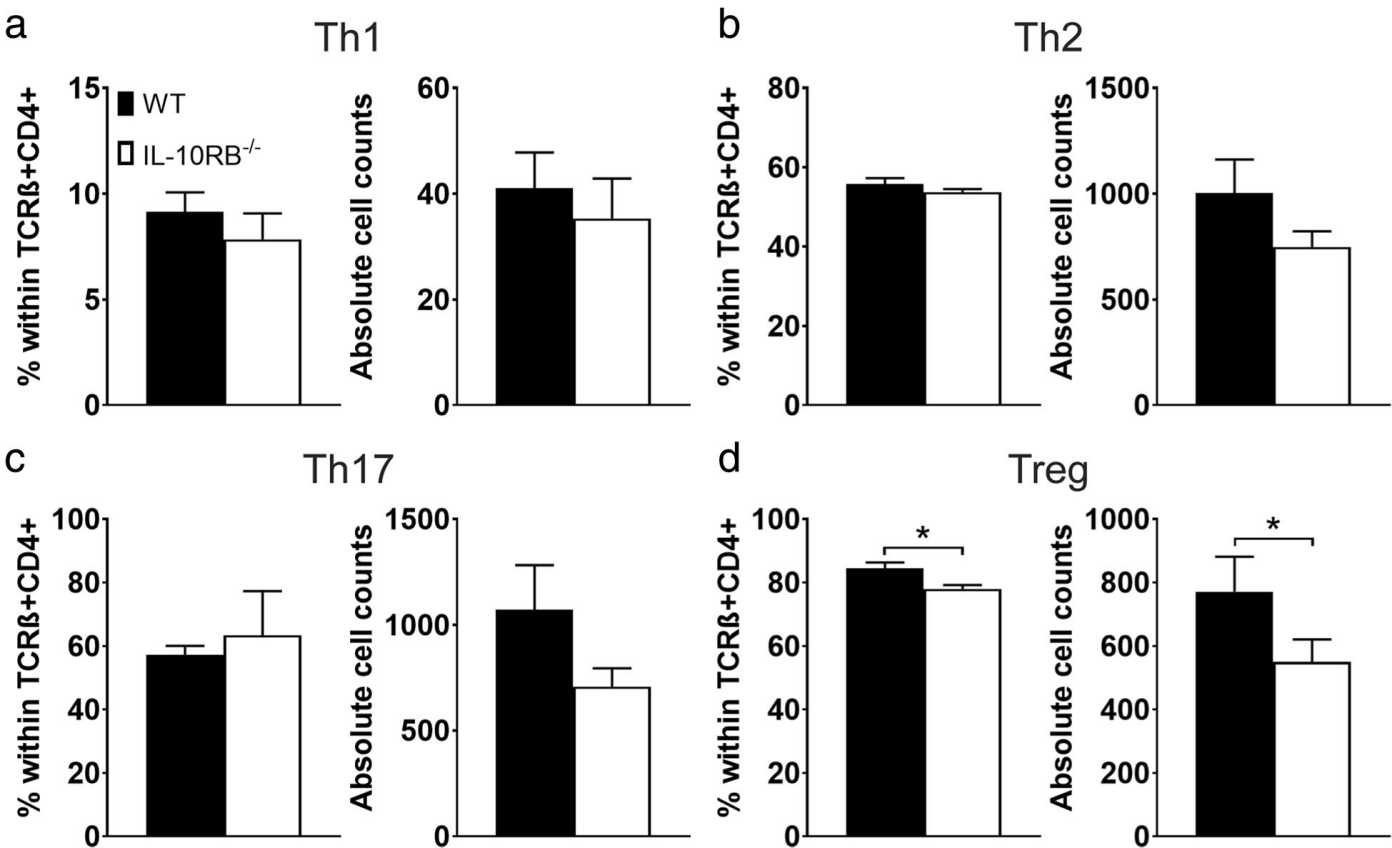
Differentiation of **a** Th1, **b** Th2, **c** Th17, and **d** Treg helper subsets in WT (black bars) and *Il10rb*^-/-^ splenocytes (white bars) stimulated with anti-CD3, anti-CD28, PMA, and ionomycin in vitro. Subsets are shown both as percentage of cells within TCRβ + CD4 + parent population as well as absolute cell counts calculated from percentages. Bars represent mean ± SEM. *p < 0.05

Flow cytometry was utilized to determine whether loss of IL-10RB affects T cell subset differentiation. Both unstimulated and in vitro stimulated splenocytes from WT and *Il10rb*^-/-^ mice were included as groups for statistical analysis; in Fig. 10, only data from stimulated cells are shown. Baseline (unstimulated) numbers of cells within T cell subsets did not differ between WT and *Il10rb*^-/-^ splenocytes and whether expressed as percent within the overall CD4+ T cell population or as absolute cell counts (data available upon request). After stimulation, there were no differences between WT and *Il10rb*^-/-^ in populations of Th1 (Fig. 10a, 9.14 ± 0.92% vs. 7.84 ± 1.22%; 41 ± 6.81 vs. 35 ± 7.54), Th2 (Fig. 10b, 55.8 ± 1.46% vs. 53.77 ± 0.72%; 1003 ± 158.8 vs. 748 ± 73.51), or Th17 cells (Fig. 10c, 57.17 ± 2.89% vs. 63.43 ± 13.82%; 1072 ± 209.3 vs. 709 ± 85.85). There was a small but significant reduction in Tregs in *Il10rb*^-/-^ compared to WT splenocytes when analyzed as percent within CD4+ T cell population (Fig. 10d, 77.93 ± 1.22% in *Il10rb*^-/-^ vs. 84.5 ± 1.76% in WT, *p* < 0.05) as well as absolute cell counts (550 ± 71.01 in *Il10rb*^-/-^ vs. 771 ± 110.6 in WT, *p* < 0.05).

## Discussion

This study demonstrates that CD3+ CD4+ T cells are indeed capable of crossing the blood-brain barrier into the FMNuc parenchyma after axotomy, where they may interact directly with CNS resident cells. It is therefore possible that immune-mediated neuroprotection and other central molecular responses to axotomy influenced by peripheral immune status are effected by local paracrine signaling from infiltrating T cells or by direct contact between T cells and CNS resident cells in the FMNuc. We demonstrate that CD4+ T cells often localize with microglial nodules, which is in agreement with other studies finding accumulation of CD3+ cells near microglial clusters after FNA [11, 28]. Microglial nodules in the FMNuc are associated with phagocytosis of dead neurons and are characterized by strong MHC class II immunoreactivity, making them potential “hotspots” for presentation of neuronal antigen and activation of infiltrating T cells [28, 29].

A previous study from our laboratory showed that both IL-10 and CD4+ T cells are necessary for FMN survival, but CD4+ T cells are not a required source of IL-10 in the FNA injury model. Interestingly, RAG-2^-/-^ mice have transiently depressed IL-10 levels in the FMNuc during the early post-injury phase which are restored by CD4+ T cell reconstitution [19]; however, this effect is modest, resolves quickly, and occurs prior to significant central T cell infiltration as described by others [11, 28]. Although it is still possible that maintenance of IL-10 expression by CNS resident cells is one mechanism by which CD4+ T cells support FMN survival, further investigation into a potential link between IL-10-mediated and adaptive immune-mediated mechanisms of neuroprotection was warranted. The data in this study expand our knowledge of the requirements for both IL-10 and CD4+ T cells in the injured FMNuc. Because overall levels of IL-10 in the FMNuc do not change in response to axotomy in the WT animal [19], induction of neuroprotective IL-10 signaling depends on increased IL-10R expression, likely on neurons and/or astrocytes [19, 28]. CD4+ T cells may be necessary for full induction of neuroprotective IL-10 signaling by triggering upregulation of IL-10R gene expression in the FMNuc, regardless of their own IL-10RB expression or IL-10 signaling capability. A recent study on immune-mediated neuroprotection in the context of chemotherapy-induced peripheral neuropathy (CIPN) bears striking similarities with our observations after FNA. Krukowski et al. demonstrated that CD8+ T cells and IL-10 are necessary for recovery of normal sensation after CIPN; however, the T cell is not the requisite source of IL-10. Rather, the presence of CD8+ T cells is necessary for upregulation of *Il10ra* mRNA in dorsal root ganglion neurons after paclitaxel-induced injury [38]. These data support a role for T cell regulation of the IL-10 signaling cascade via induction of IL-10R expression on neurons after peripheral nerve injury.

IL-10 has direct trophic effects on neurons, of which various populations have been shown to express IL-10R in vivo and in vitro [18, 19, 28, 39, 40]. Application of exogenous IL-10 to primary cortical neurons undergoing oxygen-glucose deprivation challenge suppresses apoptosis, promotes neurite outgrowth, and enhances synaptogenesis [39]. In cultured spinal cord neurons, IL-10R activation prevents glutamate-induced excitotoxicity [18]. Exogenous IL-10 treatment also improves neuron survival in vivo after spinal cord injury through regulation of pro- and anti-apoptotic factors [41]. An increase in neuronal IL-10R expression mediated by CD4+ T cells may therefore have an important role in trophic support for injured motoneurons after axotomy. The proposal that these trophic signals must arrive centrally via IL-10R signaling on the neuron cell body is congruent with a previous finding from our laboratory which showed that application of exogenous IL-10 to the peripheral nerve stump fails to restore neuron survival in either IL-10^-/-^ or RAG-2^-/-^ mice [19]. Future studies will investigate the effects of selective application of IL-10 to the central compartment after axotomy.

Despite regulating IL-10R gene expression on other cells normally, T cells that are unresponsive to IL-10 themselves via loss of the IL-10RB subunit fail to confer neuroprotection after axotomy. Although IL-10RB is considered a promiscuous receptor subunit that is shared with some interferon receptors and other IL-10 family cytokines, cells lacking IL-10RB respond normally to both type I interferons and IFNγ, but are completely unresponsive to IL-10 [32, 33, 42]. Loss of IL-10RB alone is sufficient to recapitulate the auto-inflammatory intestinal pathology observed in IL-10^-/-^ animals [33]. IL-10 directly inhibits CD28 phosphorylation in T cells and induces T cell anergy [43–47]. This loss of tolerance to self-antigen after axotomy in *Il10rb*^-/-^ CD4+ T cells could be the cause for their failure to mediate neuroprotection.

Tolerized T cells actively suppress APCs by downregulating their expression of the antigen presentation and co-stimulation molecules MHC class II, CD80, and CD86 [22]. Low CD86 is associated with tolerogenic APCs and an anti-inflammatory microglia phenotype [48–51], whereas increased microglial MHC class II and CD86 are associated with neurodegenerative and autoimmune conditions [52–55]. The *Il10rb*^-/-^ CD4+ T cells utilized in this study promoted greater expression of *Cd68*, *H2ab1*, and *Cd86*, suggesting that they may enhance microglial activation, antigen presentation, and T cell co-stimulation. Loss of IL-10 signaling in CD4+ T cells may dis-inhibit a deleterious autoimmune response mediated by microglia after injury.

MHC class I immunoreactivity is highly specific to microglial phagocytic nodule formation in the injured FMNuc [15, 16]. FNA also activates microglial expression of *C3* mRNA and the C3 receptor [56–58]. High complement, including C3, and exaggerated synaptic pruning are associated with neuronal death in CNS disease and injury [59–66]. *Il10rb*^-/-^ CD4+ T cells promoted expression of both *B2m* and *C3*, implicating T cells as modulators of microglial phagocytosis and synaptic pruning. Because synapse elimination can be either destructive or protective by shielding injured FMN from excitotoxic inputs [67], the modest increase in *B2m* and *C3* caused by WT CD4+ T cells in this study may represent a “Goldilocks zone” for beneficial synaptic elimination after FNA. However, the greater increases in *B2m* and *C3* observed after transfer of *Il10rb*^-/-^ CD4+ T cells suggest that increased synaptic elimination (or perhaps phagocytosis of stressed neurons) may enhance neuronal death after axotomy.

Other studies have demonstrated the importance of IL-10R signaling specifically in T cells. Adoptive transfer of CD4+ T cells overexpressing a dominant-negative form of IL-10RA into immunodeficient mice causes the development of spontaneous enterocolitis [68]. In mouse models and in humans, T cells nonresponsive to IL-10 escape Treg control, proliferate to a greater degree, and produce higher amounts of inflammatory cytokines such as Th17 and IFNγ, both of which activate macrophages and promote cellular autoimmunity [68, 69]. Interestingly, IL-10R signaling and downstream STAT3 phosphorylation are defective in CD4+ T cells from patients with systemic lupus erythematosus and MS, suggesting that failure of T cell suppression by IL-10 may play an important role in autoimmune disease, including in the CNS [70, 71].

The harmful role of autoreactive T cells is supported by studies finding that immunization with or without neural antigen exacerbates motoneuron loss after spinal cord injury and FNA, indicating an overall detrimental effect of enhancing adaptive immune reactivity on neuron survival [72, 73]. Those findings, as well as those described in this study, initially appear to contradict previous results from our laboratory that T cells must encounter MHC class II both peripherally and centrally in order to confer neuroprotection after axotomy [6]. However, high MHC class II expression in the absence of adequate co-stimulation may represent a mechanism by which microglia attempt to terminate autoreactive T cell responses [74]. Therefore, the requirement for dual compartment MHC class II expression for neuroprotection may actually represent the need for deletion of certain autoreactive T cell populations after axotomy.

The failure of *Il10rb*^-/-^ CD4+ T cells to promote neuroprotection also prompted the hypothesis that loss of IL-10 signaling may result in failure of differentiation of the neuroprotective Th2 subtype. Surprisingly, the numbers of Th1, Th2, and Th17 cells were not affected by loss of IL-10RB, but a decrease in the Treg population was observed. Although natural Tregs have been previously ruled out as important for neuroprotection after FNA [75], these data suggest that inducible Tregs (iTregs) may have a neuroprotective role after axotomy that is dependent on intact IL-10 signaling. However, it is important to note that interpretation of this study is limited, as T cell differentiation was elicited by non-specific stimulation of splenocytes in vitro rather than by FNA-associated antigen displayed on APCs. Furthermore, it is possible that *Il10rb*^*-/-*^ CD4+ T cells become skewed toward a particular phenotype only after entering an inflammatory microenvironment in the axotomized FMNuc. It is becoming increasingly recognized that CD4+ T cell subsets do not necessarily represent terminally differentiated cell populations, but can demonstrate considerable plasticity depending on the cytokine microenvironment [76]. Future studies will evaluate the ability of adoptively transferred *Il10rb*^-/-^ CD4+ T cells to differentiate in vivo into T cell subsets (including iTregs) after FNA.

## Conclusions

Neuroprotective CD4+ T cells infiltrate the FMNuc after axotomy and are required for full induction of central IL-10R expression. CD4+ T cells lacking IL-10RB fail to confer neuroprotection after axotomy and promote a microglial gene signature associated with enhanced antigen presentation, T cell co-stimulation, and synaptic elimination. An autoimmune response to axotomy may also be exacerbated by decreased iTreg numbers in *Il10rb*^*-/-*^ mice. These results suggest that although the degree of neuronal death in immunodeficient mice and mice reconstituted with *Il10rb*^-/-^ CD4+ T cells is comparable, the causes of FMN death are likely quite distinct. In immunodeficient mice, the neuroprotective Th2 subset is absent, and there is a blunted glial response to axotomy [4, 5]. In contrast, mice given *Il10rb*^-/-^ CD4+ T cells may exhibit dis-inhibition of a harmful autoimmune response that promotes microglial hyper-responsiveness to injury. In the spectrum of immune responses that can occur after axotomy, neither extreme is favorable, and optimum FMN survival likely depends on balanced activation of immune effectors.

## Data Availability

The datasets used and/or analyzed during the current study are available from the corresponding author on reasonable request.

## Abbreviations

APC: Antigen presenting cell
B2m: Beta-2 microglobulin
BBB: Blood-brain barrier
C3: Complement 3
CD: Cluster of differentiation
Dpo: Days post-operation
FMN: Facial motoneuron
FMNuc: Facial motor nucleus
FNA: Facial nerve axotomy
*Gapdh*: Glyceraldehyde 3-phosphate dehydrogenase
*H2ab1*: Histocompatibility 2, class II antigen A, beta 1
IBA1: Ionized calcium binding adaptor molecule 1
IL: Interleukin
IL-10R: Interleukin-10 receptor
MHC: Major histocompatibility complex
mRNA: Messenger ribonucleic acid
*Nos2*: Nitric oxide synthase 2
PBS: Phosphate buffered saline
PFA: Paraformaldehyde
PMA: Phorbol 12-myristate 13-acetate
qPCR: Quantitative polymerase chain reaction
RAG-2: Recombinase activating gene 2
RT: Reverse transcription
Th: T helper
Treg: Regulatory T cell
WT: Wild-type
